# Selective quantitative *N*-functionalization of unprotected α-amino acids using NHC-Ir(III) catalyst

**DOI:** 10.1016/j.xpro.2023.102147

**Published:** 2023-03-14

**Authors:** Beatriz Saavedra, Aitor Bermejo-López, Majken Raeder, Belén Martín-Matute

**Affiliations:** 1 Department of Organic Chemistry, Arrhenius Laboratory, Stockholm University, 106 91 Stockholm, Sweden

**Keywords:** Chemistry, Environmental Sciences

## Abstract

Unnatural amino acids are valuable building blocks with numerous applications. Here, we present a quantitative technique for accessing mono-*N*-functionalized amino acids directly from unprotected substrates using alcohols as alkylating agents and an NHC-Ir(III) catalyst. We detail specific steps for catalyst preparation and application, as well as for catalyst recycling. The protocol excludes a few amino acids (l-cysteine, l-lysine, and l-arginine) and secondary alcohols.

For complete details on the use and execution of this protocol, please refer to Bermejo-López et al. (2022).^1^

## Highlights

• Synthesis of a robust NHC-Ir(III) catalyst for selective *N*-alkylation reactions
• Mono-*N*-alkylation of unprotected chiral amino acids using alcohols as alkylating agents
• Quantitative yields and straightforward purification of *N*-modified amino acids
• Recyclability procedure for the reuse of the homogeneous NHC-Ir(III) catalyst

## Abstract (editor highlights)

Publisher’s note: Undertaking any experimental protocol requires adherence to local institutional guidelines for laboratory safety and ethics.

## Graphical abstract

**Figure:**
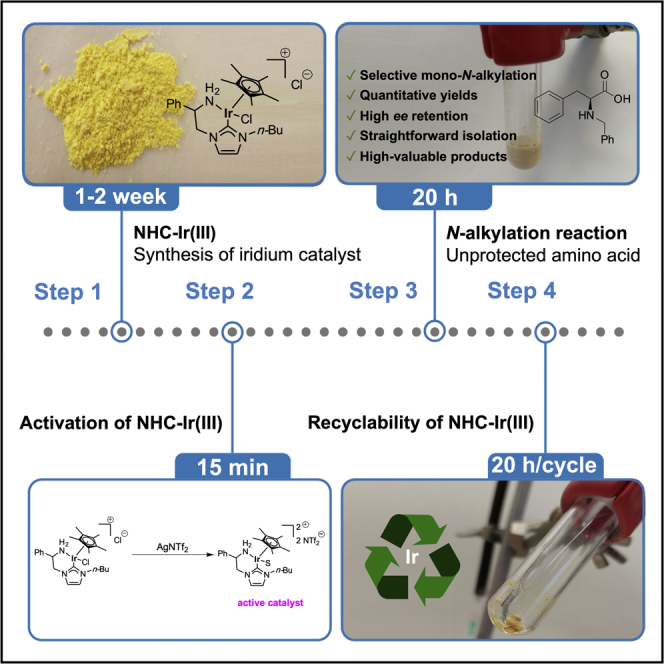

## Before you begin

### Background

*N*-Substituted amino acids are commonly used as chiral building blocks for the synthesis of pharmaceuticals,^2^^,^^3^ biodegradable surfactants^4^ and ligands for asymmetric catalysts,^5^^,^^6^ among others. However, previous synthetic methodologies to obtain these compounds from unprotected amino acids present several limitations due to the nature of these subtrates.^7^ Amino acids have limited solubility in non-polar organic solvents, and their zwitterionic nature makes them sensitive to pH changes and basic/acidic reagents.^8^^,^^9^ *N*-Alkylation of unprotected amino acids is problematic since competing esterification of the carboxylic moiety may take place. Moreover, their high polarity complicates the purification/separation from the unreacted starting materials or by-products, which is normally tackled by protection/purification/deprotection steps. This increases significantly the number of synthetic steps to obtain the target molecule, requiring longer times and unnecessary use of resources.

To this date, there is only one methodology capable of *N*-alkylating unprotected amino acids with alcohols through hydrogen borrowing strategy in good yields and excellent retention of the optical purity.^10^ However, double *N*-alkylations were obtained in most of the cases and further derivatization steps were needed to purify the products that failed to be formed in quantitative yields.

**Scheme 1:**
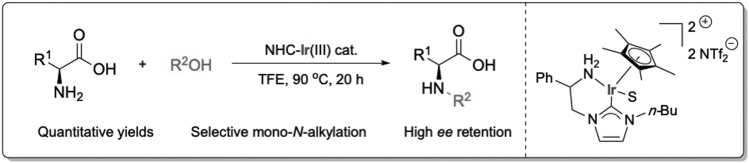
Selective and quantitative mono-*N*-alkylation of unprotected amino acids (S = solvent)

This protocol describes an iridium(III)-catalyzed selective mono-*N*-alkylation of unprotected l-phenylalanine with benzyl alcohol. The method is based in our recent work^1^ where more than 100 chiral amino acids were *N*-functionalized with outstanding efficiency and with no need for further derivatization or purification steps (Scheme 1).

The preparation of the NHC-Ir(III) catalyst is described within this protocol, as well as the mono-*N*-alkylation of l-phenylalanine. The method can be expanded to a broad scope of amino acids described in Bermejo-López et al.^1^

### Preparations

1. Dry 2,2,2-trifluoroethanol (TFE) used as the solvent for the synthesis of *N*-alkylated amino acids with activated molecular sieves (4 Å).
2. Use anhydrous solvents obtained from VAC solvent purification (when specified) for the NHC-Ir(III) catalyst synthesis and its activation prior to the *N*-alkylation reaction.

**CRITICAL:** All steps involve the use of toxic and volatile organic compounds, therefore all reactions are performed in fume hoods. Personal protection equipment is also used (gloves, goggles and lab coat).

## Key resources table

**Table:** 

REAGENT or RESOURCE	SOURCE	IDENTIFIER
**Chemicals, peptides, and recombinant proteins**		

Ag_2_O +99%	Strem Chemicals	CAS 20667-12-3
AgNTf_2_ 99.9%	Ambeed	CAS 189114-61-2
Ammonia solution 25% (NH_4_OH)	Honeywell Fluka	CAS 1336-21-6
Benzyl alcohol 99%	Merck	CAS 100-51-6
2-Bromoacetophenone 98%	Alfa Aesar	CAS 200-724-9
Celite® 545	Honeywell Fluka	CAS 68855-54-9
[Cp∗IrCl_2_]_2_ 98%	Strem Chemicals	CAS 12354-84-6
Ethanol 99.5%	KiiltoClean AB	CAS 64-17-5
HCl solution (0.5 M in methanol)	Acros	CAS 7647-01-0
Hydroxylamine hydrochloride 99%	Fluka	CAS 5470-11-1
Imidazole 99%	Alfa Aesar	CAS 288-32-4
l-Phenylalanine ≥98%	Sigma-Aldrich	CAS 63-91-2
Molecular sieves (4 Å)	Alfa Aesar	CAS 70955-01-0
*n*-Butylchloride 99%	Acros	CAS 109-69-3
Pd/C (10 wt%)	Sigma-Aldrich	CAS 7440-05-3
Pyridine	VWR	CAS 110-86-1
Silica 60A 35–70 micron	Fischer Chemical	CAS 7631-86-9
TMSCHN_2_ (2 M in Et_2_O)	Sigma-Aldrich	CAS 18107-18-1
Triethylamine ≥99.5%	Sigma-Aldrich	CAS 121-44-8
2,2,2-Trifluoroethanol (TFE) 99%	Apollo Scientific	CAS 75-89-8
Water	Deionized water tap (lab)	N/A

**Other**		

Caps for microwave vials (crimp caps)	DWK Life Sciences	Cat#16656872
Hamilton syringe (50 μL)	Rettberg	Cat#190007303
Microwave vials	Biotage	N/A
Round-bottom flask (100 mL)	Rettberg	Cat#134020224
Round-bottom flask (250 mL)	Rettberg	Cat#134020236
Stirring/heating plate	IKA	Cat#0003810000

## Materials and equipment

Nuclear magnetic resonance (NMR) spectra were recorded at 400 or 500 MHz for ^1^H NMR, and at 100 or 125 MHz for ^13^C NMR, on a Bruker 400 and on a Bruker AV 500 spectrometer. ^1^H NMR spectra were recorded using a relaxation delay T1 = 5 s (important integral regions of spectra with T1 > 5 s were equal to integral regions when T1 = 5 s). High-resolution mass spectra (HRMS) were obtained on a Bruker MicroTOF ESI-TOF spectrometer. Enantiomeric excess was determined using SFC-DIAD 250 mm Chiralcel or Chiralpak columns, CO_2_/CH_3_OH, using chiral stationary phases. Hydrogenation reaction performed in Parr Stainless Steel High Pressure Reactor Autoclave 1 L.

## Step-by-step method details

### NHC-Ir(III) pre-catalyst synthesis

**Timing: 1–2 weeks**

**Timing: 4 h (for step 1)**

**Timing: 1–2 days (for step 2)**

**Timing: 1–2 days (for step 3)**

**Timing: 3 days (for step 4)**

**Timing: 2 days (for step 5)**

**Timing: 3 days (for step 6)**

**Scheme 2:**
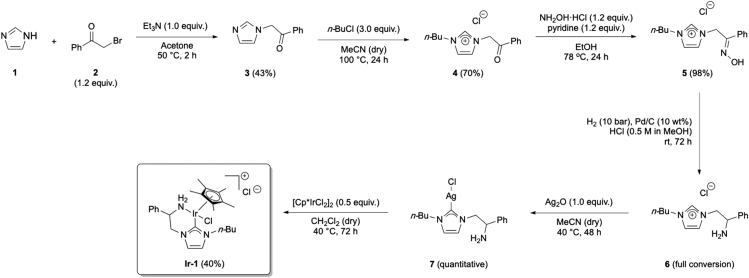
Synthesis of iridium pre-catalyst complex (**Ir-1**)

The synthesis of NHC-Ir(III) pre-catalyst (**Ir-1**) is shown in Scheme 2. From imidazole (**1**), two consecutive alkylations, first with 2-bromoacetophenone (**2**) followed by *n*-butyl chloride, gave imidazolium salt **4**. Oxime **5** is obtained in nearly quantitative yield in the following step, which is then reduced using H_2_ in the presence of Pd/C. Imidazolium salt **6** is used in the next step without further purification. A silver carbene is formed upon reaction with Ag_2_O, which afterward is transmetalated with [Cp∗IrCl_2_]_2_ to give the thermally and air stable pre-catalyst iridium complex (**Ir-1**).

**Figure 1:**
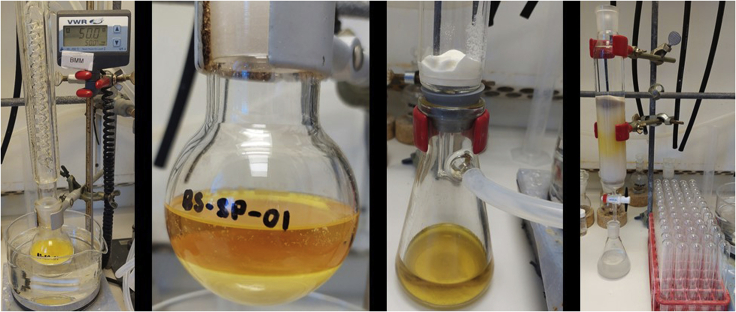
Reaction and appearance of imidazole **3** during synthesis and purification stages From left to right: reaction set-up under reflux; reaction mixture after completion and termination of stirring; filtration of the crude mixture; purification by column chromatography.

**Figure 2:**
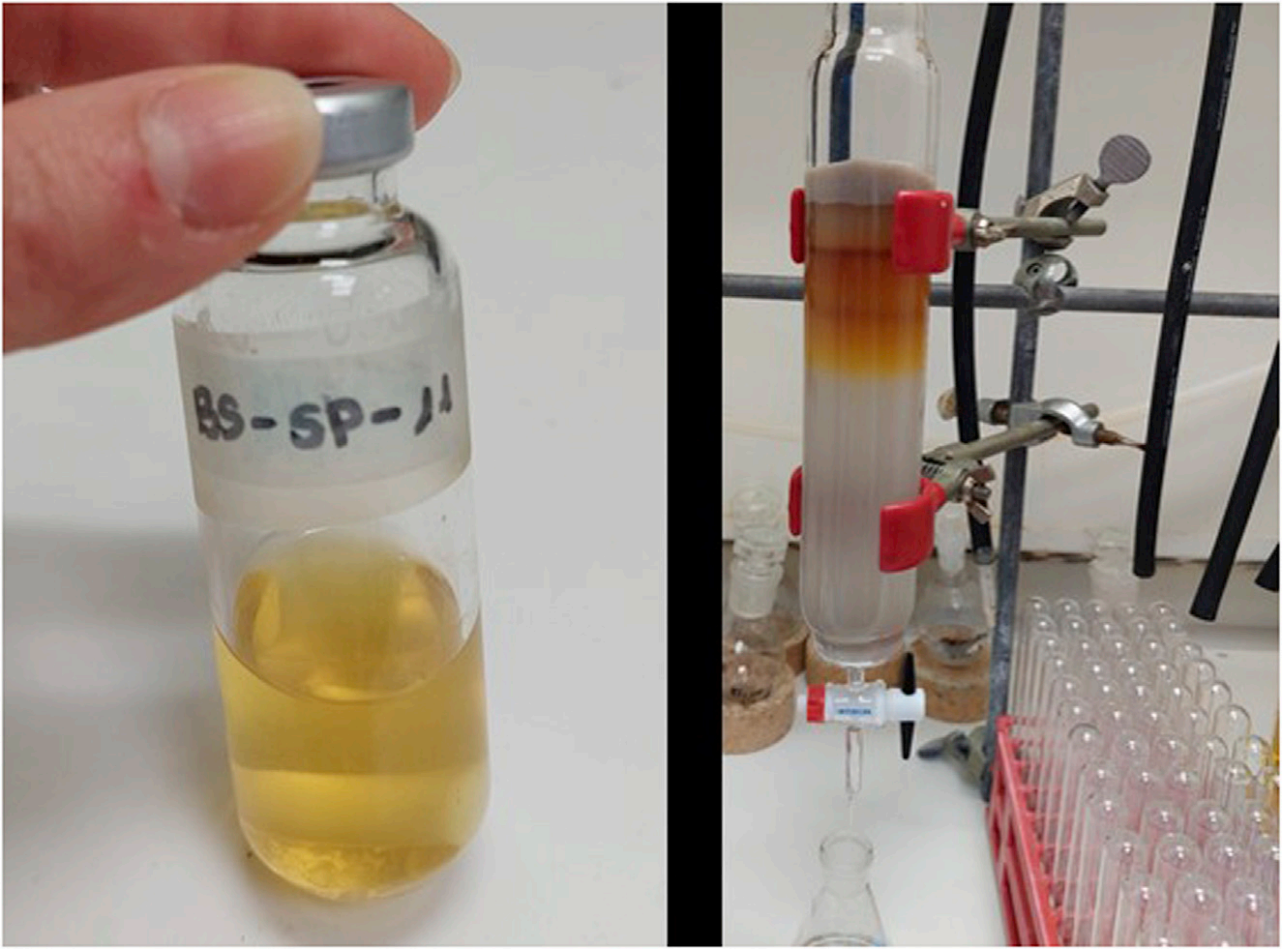
Reaction and appearance of the product during different stages in the synthesis of imidazolium salt **4**: reaction vessel before the reaction and flash chromatography purification step (from left to right)

**Figure 3:**
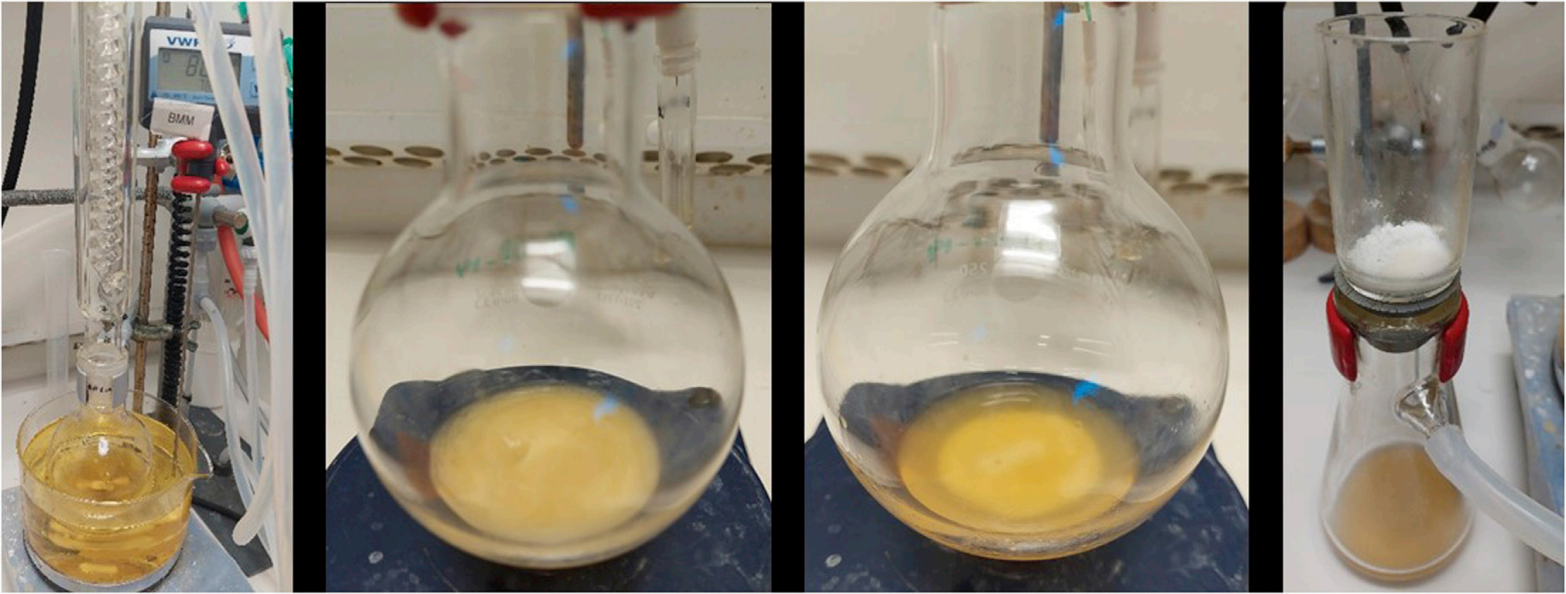
Reaction and appearance of the product during different stages in the synthesis of oxime **5** From left to right: reaction set-up under reflux; cloudy suspension formed after CH_2_Cl_2_ addition under stirring; precipitation of unwanted salts when the stirring is stopped; filtration step to remove the unwanted salts.

**Figure 4:**
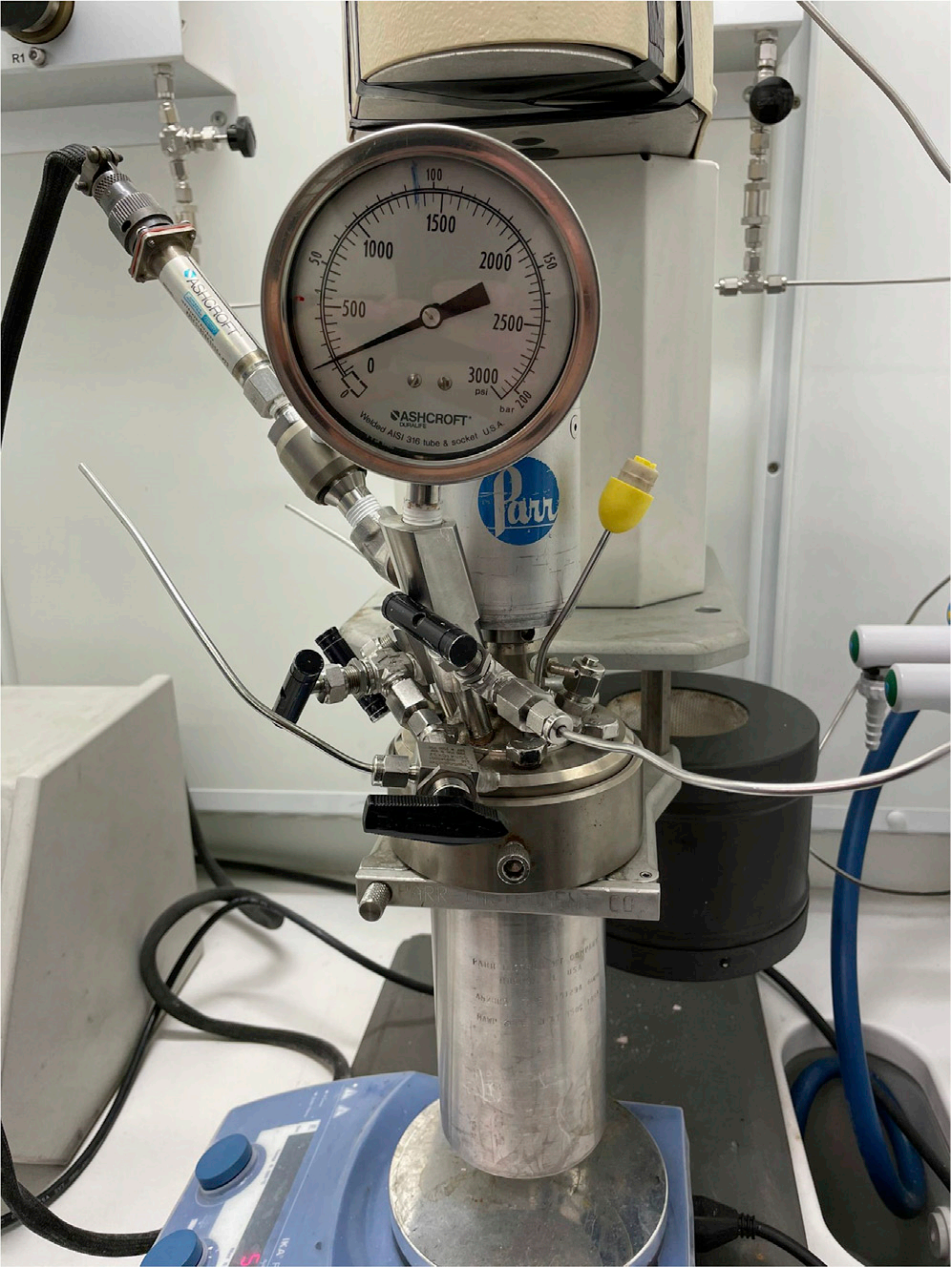
Set-up of the high-pressure hydrogenation apparatus of synthesis of compound **6**

1. Synthesis of imidazole **3** (Figure 1).
  a. Weight out imidazole (1.36 g, 20 mmol) and 2-bromoacetophenone (4.77 g, 24 mmol, 1.2 equiv.) and add to a 100 mL round-bottom flask.
  b. Add 50 mL of acetone and Et_3_N (2.8 mL, 24 mmol, 1.0 equiv.) at room temperature with constant stirring (300–400 rpm; 20°C–23°C).
  c. Introduce the mixture in a pre-warmed oil bath at 50°C (under reflux) for 2 h.
    ***Note:*** a white precipitate immediately forms.
  d. Cool down the mixture to room temperature (20°C–23°C) and filter-off the white precipitate; wash it with acetone (20 mL × 3).
    ***Note:*** the white solid can be analyzed by ^1^H NMR (DMSO-d_6_) observing a mixture of the triethylammonium salt and the corresponding salt derived from the alkylated product.
  e. Dry the remaining solution containing the desired product **3** over MgSO_4_ (2 g) and evaporate the solvent under reduced pressure in the rotavapor (40°C, 500 mbar).
  f. Purify by column chromatography using acetone as eluent. Imidazole **3** is obtained as a yellowish solid (1.61 g, 43% yield).
    ***Note:*** analyze the crude by TLC with acetone as eluent. The product has an R_f_ = 0.5.
    i. Dry loading (dissolve compound **3** in the minimum amount of acetone (20–30 mL), add silica (0.5–1 g) to the mixture, remove the solvent under reduced pressure and add the silica containing compound **3** to the column) to a silica gel column (⌀ = 4 cm, height of silica added = 15 cm).
      ***Note:*** silica is loaded and packed in the column using acetone as solvent. No airflow is used once the product is loaded in the column.
    ii. Use TLC (acetone as eluent) with UV-light detection (254 nm) to analyze the fractions.
    iii. Collect the fractions containing the product (R_f_ = 0.5).
      ***Note:*** discard the first dark yellow fraction. After this, the product comes out (be careful in the last tubes taken since imidazole 1 comes next).
      ^1^H NMR (400 MHz, CDCl_3_, 298 K): δ = 7.99 (dd, 2H, *J* = 8.6, 6.3 Hz), 7.64–7.69 (m, 1H), 7.52–7.59 (m, 3H), 7.15 (t, 1H, *J* = 7.5 Hz), 6.96 (t, 1H, *J* = 7.5 Hz), 5.43 (s, 2H) ppm; ^13^C NMR (100 MHz, CDCl_3_, 298 K): δ = 191.5, 138.1, 134.4, 134.2, 129.3, 129.1, 128.0, 120.3, 52.5 ppm; HRMS-ESI calcd for C_11_H_11_N_2_O [M + H]^+^: 187.0866. Found: 187.0866.

2. Synthesis of imidazolium salt **4** (Figure 2).
  a. In a 30 mL microwave tube, dissolve compound **3** (1.7 g, 9.13 mmol) in dry acetonitrile (7 mL).
  b. To the same vessel, add *n-*butylchloride (2.9 mL, 27.39 mmol, 3.0 equiv.).
  c. Close the vessel with the corresponding aluminum cap and heat it at 100°C for 24 h.
    ***Note:*** oil bath already warmed at the corresponding temperature.
  d. Cool down the mixture to room temperature (20°C–23°C).
  e. Remove both the solvent and excess of *n*-butylchloride under reduced pressure in a rotavapor (40°C, 200 mbar).
  f. Purify by column chromatography using CH_2_Cl_2_/MeOH as eluent (gradient from 20:1–5:1), to obtain imidazolium salt **4** as a thick oil (1.32 g, 70% yield).
    ***Note:*** analyze the crude by TLC with CH_2_Cl_2_/MeOH (5:1) as eluent. The product has an R_f_ = 0.5.
    i. Dry loading to a silica gel column (⌀ = 4 cm, height of silica added = 17 cm).
      ***Note:*** silica is loaded and packed in the column using CH_2_Cl_2_/MeOH (20:1) as solvent. No airflow is used once the product is loaded in the column.
    ii. Use TLC (CH_2_Cl_2_/MeOH 5:1 as eluent) with UV-light detection (254 nm) to analyze the fractions.
    iii. Collect the fractions containing the product (R_f_ = 0.5).
      ***Note:*** start with CH_2_Cl_2_/MeOH 20:1 until the first dark brown fraction comes out, then change to CH_2_Cl_2_/MeOH 10:1 and continue until you do not see any impurity with high R_f_. Finally, use CH_2_Cl_2_/MeOH 5:1 to elute the product.
      ^1^H NMR (400 MHz, CDCl_3_, 298 K): δ = 10.42 (s, 1H), 8.07 (dd, 2H, *J* = 8.5, 6.2 Hz), 7.61–7.65 (m, 2H), 7.48–7.59 (m, 2H), 7.34–7.35 (m, 1H), 6.43 (s, 2H), 4.23–4.27 (m, 2H), 1.86–1.94 (m, 2H), 1.36–1.42 (m, 2H), 0.94–1.0 (m, 3H) ppm; ^13^C NMR (100 MHz, CDCl_3_, 298 K): δ = 190.9, 138.6, 134.8, 133.5, 129.2, 128.7, 124.2, 120.8, 55.5, 50.0, 32.0, 19.5, 13.4 ppm; HRMS-ESI calcd for C_15_H_19_N_2_O [M-Cl]^+^: 243.1492. Found: 243.1492.

3. Synthesis of oxime **5** (Figure 3).
  a. In a 250 mL round-bottom flask, dissolve imidazolium salt **4** (2.4 g, 8.7 mmol) in 60 mL of ethanol.
  b. Add NH_2_OH·HCl (0.72 g, 10.4 mmol, 1.2 equiv.).
  c. Add pyridine (0.84 mL, 10. 4 mmol, 1.2 equiv.) to the reaction mixture.
  d. Reflux the mixture (oil bath temperature of 78°C) for 24 h.
    ***Note:*** oil bath already warmed up at the corresponding temperature.
  e. Remove the solvent under reduced pressure in a rotavapor (40°C, 170 mbar).
    ***Note:*** full conversion can be confirmed by ^1^H NMR (MeOD-d_4_).
  f. Add H_2_O (3 mL) and a solution of NH_3_ in H_2_O (25%; 1.62 mL, 1.2 equiv.) dropwise while stirring (10–15 min; 300–400 rpm).
  g. Remove the solvent at reduced pressure in a rotavapor.
  h. Dissolve the oily product **5** in the minimum amount of methanol (5–10 mL).
  i. Add CH_2_Cl_2_ until the solution becomes cloudy (ca 7–10 mL).
  j. Store the mixture in the freezer (−18°C) for 15–30 min.
  k. Filter-off the solution (containing the desired product) to remove the unwanted white solid. Wash the solid with CH_2_Cl_2_ (15 mL × 3).
  l. Concentrate the filtrate under reduced pressure in a rotavapor. Repeat the process (from step h to k) 4 times to yield 2.5 g of oxime **5** (98% yield).
    ^1^H NMR (400 MHz, CD_3_OD, 298 K, E/Z mixture): δ = 9.11–9.12 (m, 1H), 7.71–7.73 (m, 1H), 7.57–7.63 (m, 2H), 7.41–7.44 (m, 2H), 5.60–5.37 (m, 2H), 4.18–4.26 (m, 2H), 1.77–1.89 (m, 2H), 1.19–1.41 (m, 2H), 0.92–1.01 (m, 3H) ppm; ^13^C NMR (100 MHz, CD_3_OD, 298 K): δ = 150.0, 149.6, 136.7, 136.6, 133.2, 130.2, 129.5, 129.4, 128.6, 128.2, 128.0, 126.0, 123.0, 122.4, 52.1, 49.3, 42.3, 31.7, 31.6, 18.9, 18.8, 12.3 ppm; HRMS-ESI calcd for C_15_H_20_N_3_O [M-Cl]^+^: 258.1601. Found: 258.1637.

4. Synthesis of imidazolium salt **6** (Figure 4).
  a. Dissolve oxime **5** (2.5 g, 8.5 mmol) in a solution of HCl in methanol (0.5 M, 15 mL) in a flat-bottom cylindrical reaction vessel (recipient for the hydrogenation reactor) and purge with argon.
  b. Add Pd/C (10 wt%, 200 mg, 0.19 mmol of Pd, 2.2 mol% of Pd).
  c. Place the vessel into a high-pressure hydrogenation apparatus (Parr Stainless Steel High Pressure Reactor Autoclave 1 L).
  d. Purge the reactor three times with argon, and then fill it with H_2_ (10 bar).
  e. Stir the reaction at room temperature for 72 h (500 rpm; 20°C–23°C).
  f. Release the pressure of hydrogen.
  g. Filter the mixture through a path of Celite®, and wash with MeOH (100 mL).
  h. Remove the solvent under reduced pressure in a rotavapor.
  i. Analyze the crude mixture by ^1^H NMR (MeOD-*d*_*4*_). Full conversion to imidazolium salt **6** is observed, which is used without further purification for the next step.

^1^H NMR (400 MHz, CD_3_OD, 298 K): δ = 8.97 (s, 1H), 7.63–7.64 (m, 2H), 7.43–7.48 (m, 5H), 4.60–4.81 (m, 3H), 4.15–4.19 (m, 2H), 1.75–1.82 (m, 2H), 1.21–1.27 (m, 2H), 0.93–0.97 (m, 3H) ppm; ^13^C NMR (100 MHz, CD_3_OD, 298 K): δ = 137.0, 137.6, 128.3, 124.3, 123.9, 56.3, 54.5, 50.7, 33.0, 32.8, 20.3, 13.7 ppm; HRMS-ESI calcd for C_15_H_22_N_3_ [M-Cl]^+^: 244.1808. Found: 244.1805.

**CRITICAL:** Suspending the Pd/C catalyst in the hydrogenation solution containing oxime **5** should be done under inert atmosphere. Further, Pd/C can spark spontaneously and may ignite on exposure to air after the hydrogenation reaction. Therefore, the reactor must be purged with inert atmosphere before is opened to air at the end of the reaction. The hydrogenation reaction must be performed inside a fume hood and the pressure of hydrogen released slowly.

5. Synthesis of silver carbene **7** (Figure 5).
  a. In a 30 mL microwave tube covered with aluminum foil, add imidazolium salt **6** (250 mg, 0.89 mmol), and cap the tube with a septum and purge with argon.
  b. Then, add 20 mL of dry acetonitrile and Ag_2_O (208 mg, 0.89 mmol, 1.0 equiv.).
    ***Note:*** avoid exposure to light as much as possible in all the steps. The septum of the tube is removed to add Ag_2_O. The septum is put back and an additional purge with argon must be done. The septum is then exchanged fast by a crimp cap.
  c. Stir the mixture (300–400 rpm) under argon atmosphere at 40°C for 48 h.
  d. Filter the mixture through a pad of Celite® (around 10 cm high, in the dark). Wash the Celite® column with CH_2_Cl_2_ (100 mL).
  e. Collect the combined solutions in a flask covered with aluminum foil.
  f. Remove the solvent at reduced pressure in a rotavapor.
    ***Note:*** keep the flask covered with aluminum foil.
  g. Confirm the formation of the silver carbene by ^1^H NMR (CDCl_3_).
  h. Use directly silver carbene **7** for the next step without further purification. Troubleshooting 1.

**Figure 5:**
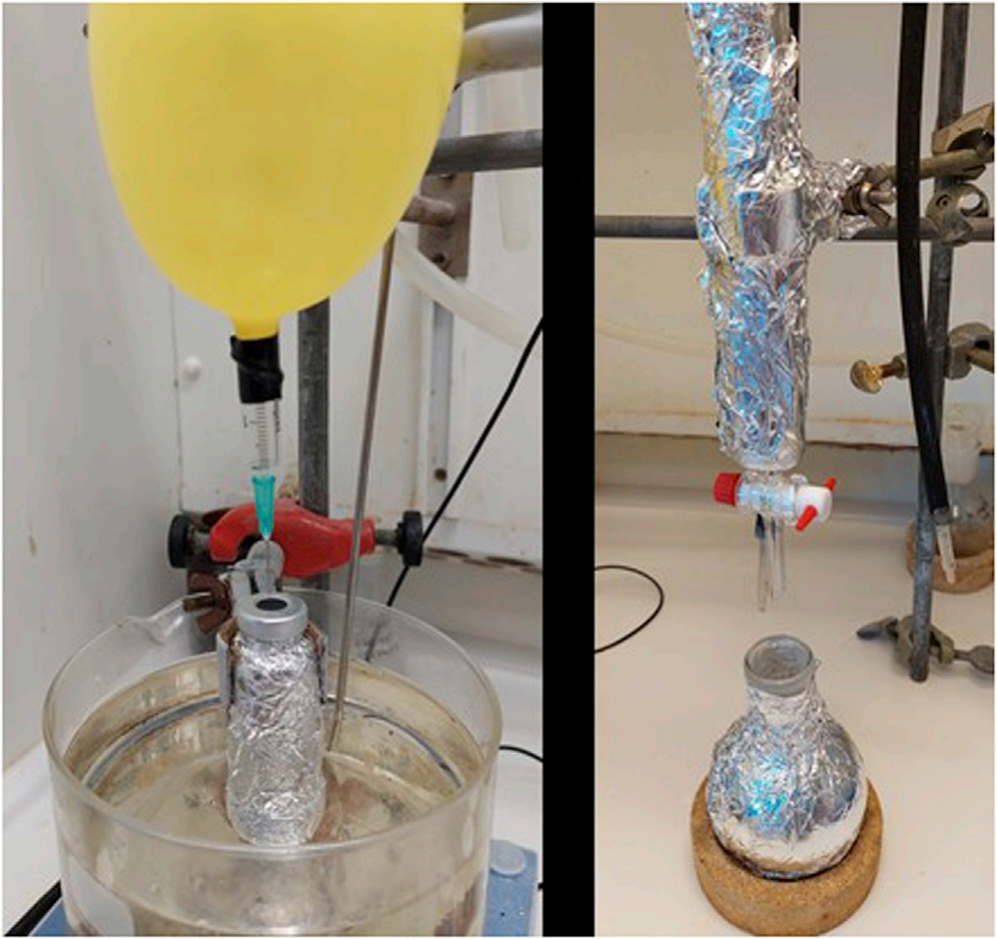
Reaction and set-up of the different stages during the synthesis of silver carbene **7**: reaction set-up and filtration through celite step (from left to right)
6. Synthesis of **Ir-1** pre-catalyst.
  a. Dissolve, silver carbene **7** obtained in the previous step, in dry CH_2_Cl_2_ (20 mL) and transfer it into a 30 mL microwave tube covered with aluminum foil and previously purged with argon.
  b. Add [Cp∗IrCl_2_]_2_ (358 mg, 0.45 mmol, 0.5 equiv.) to the reaction vessel.
  c. Stir the reaction (300–400 rpm) under argon at 40°C for 72 h.
  d. Filter the reaction mixture through a pad of Celite® and wash the Celite® with 100 mL of CH_2_Cl_2_.
    ***Note:*** after the reaction, exposure to light is no longer an issue.
  e. Evaporate the combined solutions at reduced pressure in a rotavapor.
  f. Purify iridium complex **Ir-1** by column chromatography using CH_2_Cl_2_/MeOH as eluent (gradient 20:1–10:1).
    ***Note:*** analyze the crude by TLC with CH_2_Cl_2_/MeOH (10:1) as eluent. The product has R_f_ = 0.2.
    i. Load the iridium crude mixture dissolved in the minimum amount of CH_2_Cl_2_ (2–5 mL) with a pipette to a silica gel column (⌀ = 4 cm, height of silica added = 17 cm).
      ***Note:*** silica is loaded and packed in the column using CH_2_Cl_2_/MeOH (20:1) as solvent. No airflow is used once the product is loaded in the column.
    ii. Use TLC (CH_2_Cl_2_/MeOH 10:1 as eluent) with UV-light detection to analyze the fractions.
    iii. Collect the fractions containing the product (R_f_ = 0.2).
      ***Note:*** start with CH_2_Cl_2_/MeOH 20:1 (300 mL, three times), then use CH_2_Cl_2_/MeOH 10:1 until the end. The two yellowish and orange first fractions of the column (Figure 6 -3^rd^ picture) should be discarded. Ir-1 comes out in the last yellow fraction of the column.

**Figure 6:**
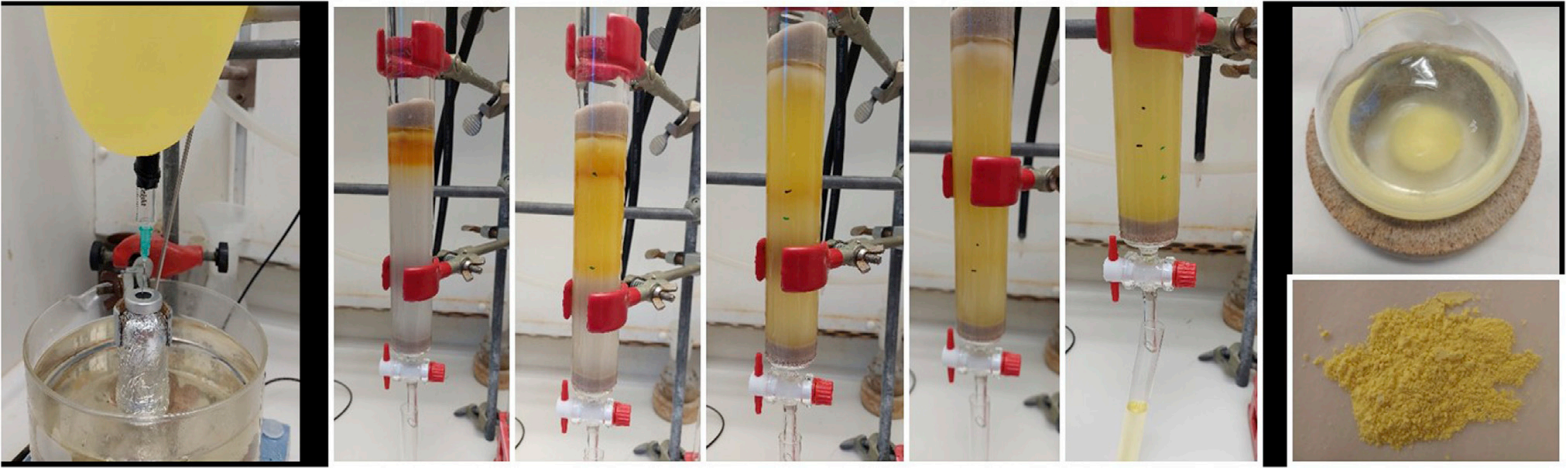
Synthesis and purification of **Ir-1**, from left to right: Reaction set-up under argon; purification by column chromatography; precipitation with pentane and **Ir-1** complex after evaporation of the solvent
  g. Remove the solvent of the combined fractions under reduced pressure in the rotavapor.
  h. Dissolve the residue with the minimum amount of CH_2_Cl_2_ (5–10 mL) and add pentane until a yellow precipitate is formed.
  i. Remove the solvent by decantation and dry the solid **Ir-1** under high vacuum. **Ir-1** is obtained as a yellow solid (215 mg, 40% yield).
    ***Note:*** if the iridium catalyst is not pure enough repeat step h to i, until a pure complex is obtained (check by ^1^H NMR spectroscopy in CDCl_3_). Troubleshooting 2.
    ^1^H NMR (400 MHz, CDCl_3_, 298 K): δ = 8.37–8.42 (m, 1H), 7.02–7.12 (m, 5H), 6.86 (d, 1H, *J* = 1.6 Hz), 6.52 (d, 1H, *J* = 1.6 Hz), 5.50–5.52 (m, 1H), 4.43–4.49 (m, 1H), 3.88–4.09 (m, 3H), 3.39 (d, 1H, *J* = 7.7 Hz), 1.77–1.99 (m, 17 H), 1.54–1.40 (m, 2H), 1.07 (t, 3H, *J* = 7.5 Hz) ppm; ^13^C NMR (100 MHz, CDCl_3_, 298 K): δ = 155.2, 137.6, 128.6, 127.8, 126.7, 123.7, 119.7, 90.5, 57.7, 53.5, 49.9, 33.3, 20.0, 13.9, 9.9 ppm; HRMS-ESI calcd for C_25_H_36_ClIrN_3_ [M-Cl]^+^: 606.2214. Found: 606.2240.

### NHC-Ir(III) activation

**Timing: 15–30 min**

**Scheme 3:**
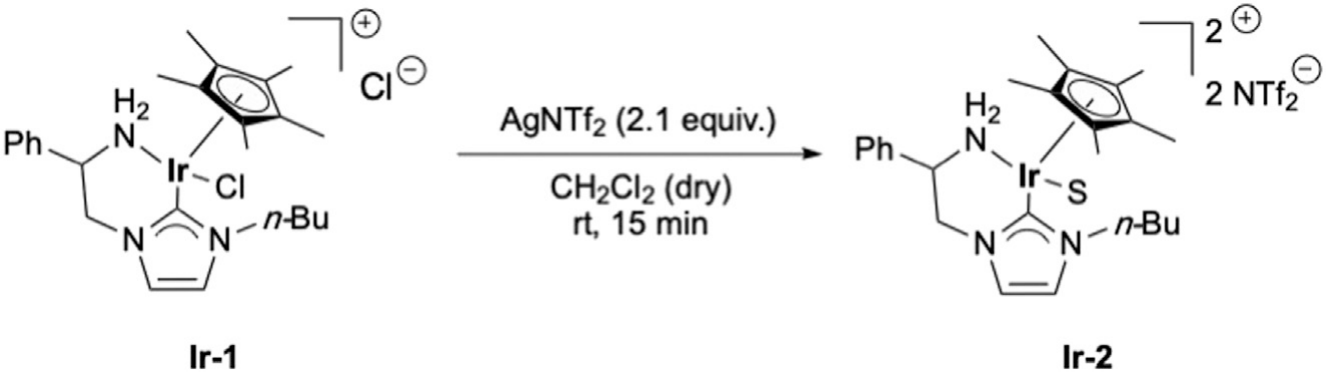
Activation of **Ir-1** complex (S = Solvent)

Prior to the mono-*N*-alkylation reaction of unprotected amino acids, the **Ir-1** pre-catalyst is activated (Scheme 3).

**Figure 7:**
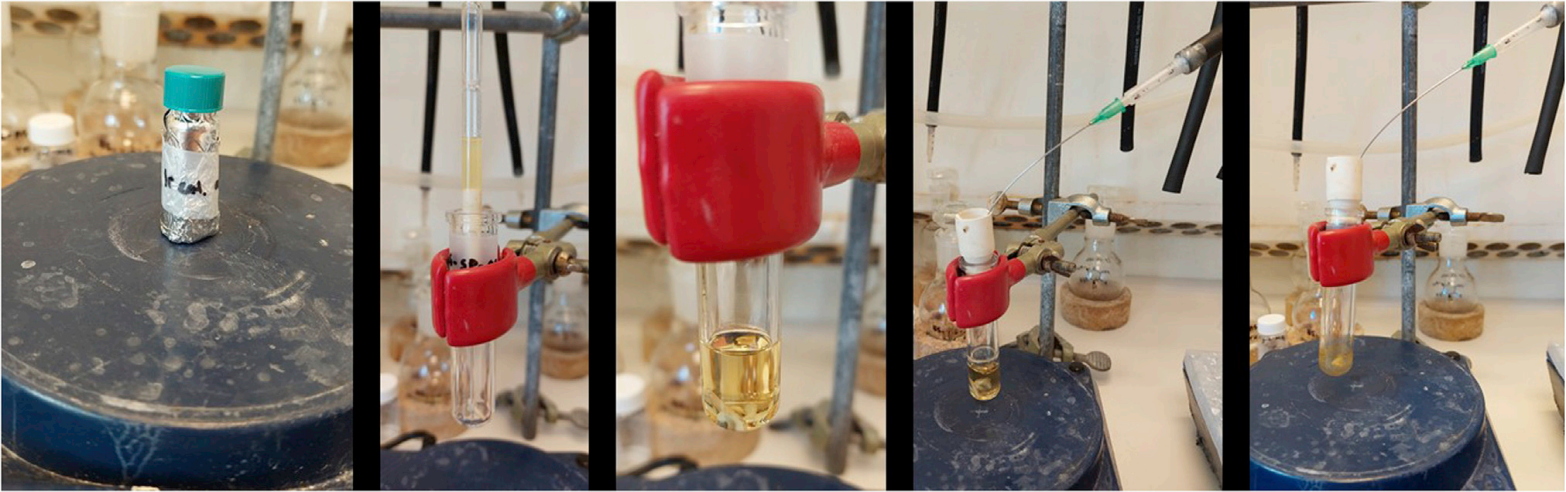
Activation procedure of **Ir-1** complex to form **Ir-2**, from left to right: reaction with AgNTf_2_; filtration through Celite®; solution after filtration; solvent drying procedure and result

***Note:*** important to remove all the CH_2_Cl_2_.

7. Activation of **Ir-1** complex (Figure 7).
  a. In a 5 mL vial covered with aluminum foil, add **Ir-1** complex (3.2 mg, 0.005 mmol) and AgNTf_2_ (4.2 mg, 0.011 mmol, 2.1 equiv.).
  b. Add 1 mL of dry CH_2_Cl_2_.
  c. Stir the reaction mixture at room temperature for 15 min (300–400 rpm; 20°C–23°C).
  d. Filter-off the mixture, directly into a 10 mL microwave tube, through a pad of Celite® to remove the generated AgCl salt (wash with another 1–2 mL of CH_2_Cl_2_).
  e. Evaporate the solvent under vacuum and use the active catalyst **Ir-2** for the mono-*N-*alkylation of unprotected amino acids.

**CRITICAL:** AgNTf_2_ salt is highly hygroscopic and sensitive to light, it must be stored in a desiccator and protected from light. Do not keep it in the fridge/freezer.

### Mono-*N*-alkylation of unprotected amino acids

**Timing: 20 h**

**Scheme 4:**
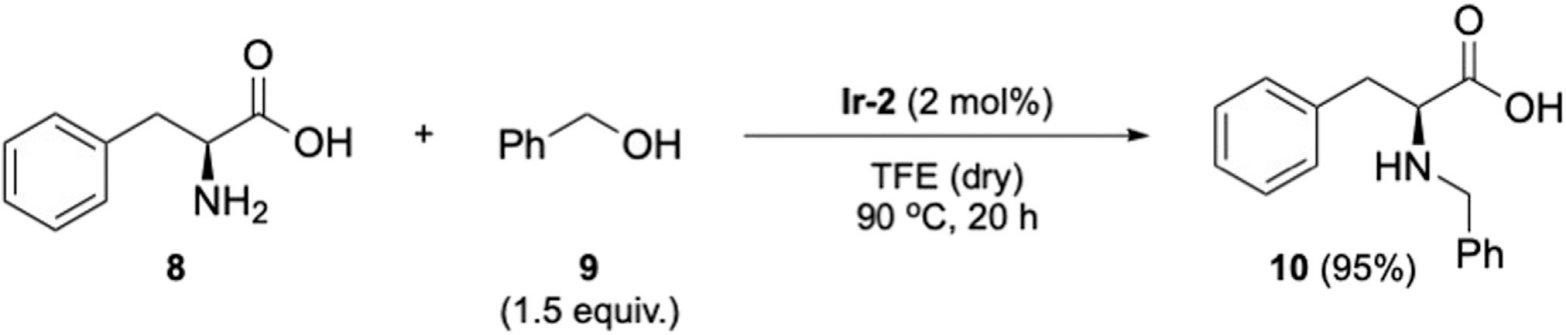
Mono-N-alkylation reaction of unprotected amino acids catalyzed by **Ir-2**

**Figure 8:**
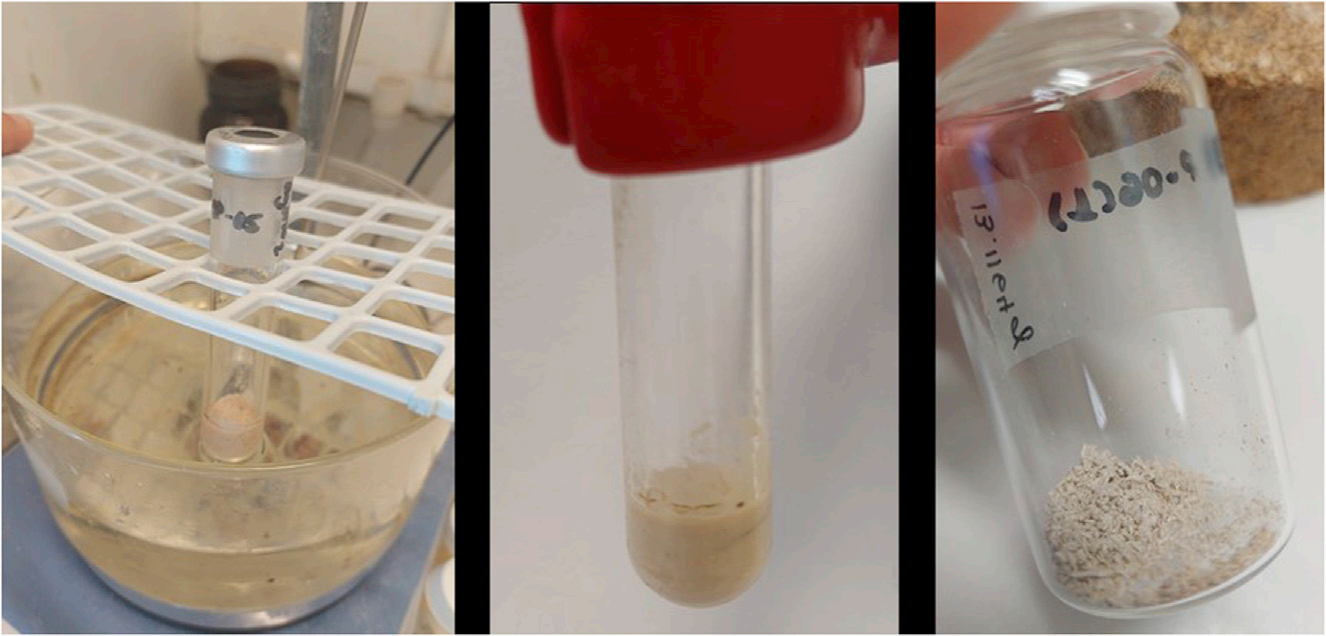
Reaction and appearance of the product **10** during different stages of its synthesis: reaction set-up, reaction completed and compound **10** as-synthesized

8. Mono-*N*-alkylation of l-phenylalanine catalyzed by NHC-Ir(III) complex (**Ir-2**, Scheme 4, Figure 8).
  a. In the same microwave vial impregnated with **Ir-2** catalyst, add l-phenylalanine (**8**; 41.3 mg, 0.25 mmol).
  b. Then, add TFE (1 mL) and benzyl alcohol (**9**; 38 μL, 0.37 mmol, 1.5 equiv.).
    ***Note:*** The reaction does not require inert atmosphere.
  c. Seal the microwave vial and stir the mixture at 90°C for 20 h.
    ***Note:*** if after a few hours an off-white precipitate does not form, the activation of Ir-1 failed. Troubleshooting 3.
  d. Cool down the vial to room temperature (20°C–23°C).
  e. Remove the solvent under reduced pressure in a rotavapor.
  f. Add diethyl ether (3 mL) to the crude and place the mixture in an ultrasonic bath for 5 min (42 kHz).
  g. Remove diethyl ether by decantation, and repeat steps f to g 3 times.
  h. After drying the solid, add MeOH (1 mL) to remove the remaining/occluded diethyl ether. Then, evaporate the solvent under reduced pressure in a rotavapor.
  i. Product *N*-benzyl l-phenylalanine **10** is isolated as a white solid (60.9 mg, 95% yield). No further purification steps are needed.
    i. SFC analysis: to analyze the ee of the product a derivatization of product 10 is performed.
    ii. Derivatization procedure: to a stirred solution of amino acid 10 (0.25 mmol) in toluene/MeOH (1:1), add TMSCHN2 (0.5 mmol, 2 M in diethyl ether). Stir the mixture for 1 h at room temperature (20°C–23°C), and concentrate the mixture under reduced pressure in a rotavapor. Quantitative conversion into the esterified amino acid is obtained. The product is directly analyzed (step iii).
    iii. SFC conditions: 5% MeOH, IF column, 0.8 mL min-1, UV 230 nm [tr = 7.38 min (major), tr = 9.53 min (minor)] – 98% ee.
      ^1^H NMR (400 MHz, CD_3_OD-KOH, 298 K): δ = 7.18–7.29 (m, 10H), 3.76 (d, 1H, *J* = 12.8 Hz), 3.56 (d, 1H, *J* = 12.8Hz), 3.34 (m, 1H), 3.02 (dd, 1H, *J* = 13.4 Hz, *J* = 6.3 Hz), 2.85 (dd, 1H, *J* = 13.4 Hz, *J* = 6.3 Hz) ppm; ^13^C NMR (100 MHz, CD_3_OD-KOH, 298 K): δ = 179.9, 139.7, 139.0, 129.1, 128.0, 127.9, 127.8, 126.5, 125.8, 64.9, 51.7, 39.7 ppm; HRMS-ESI calcd for C_16_H_17_NNaO_2_ [M + Na]^+^: 278.1151. Found: 278.1155.

### Recyclability of NHC-Ir(III) catalyst

**Timing: 20 h/per cycle**

**Figure 9:**
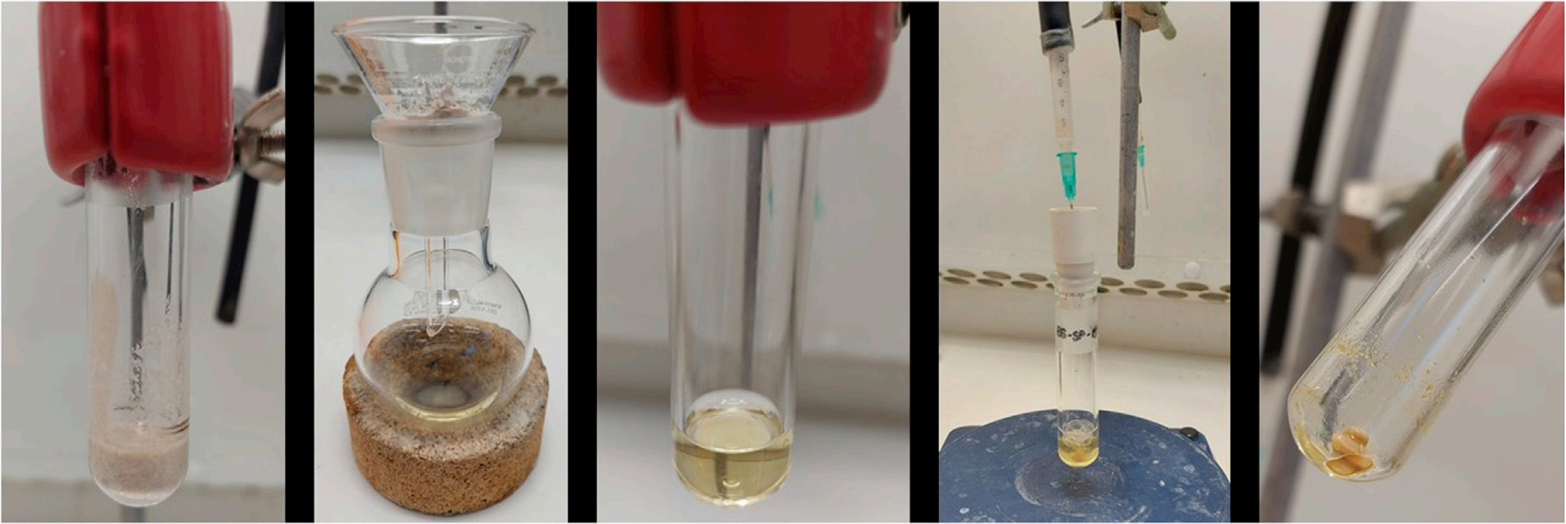
Recyclability of **Ir-2** steps, from left to right: alkylation reaction completed; filtration of product **10** (solid) and washing of **10** with Et_2_O; recovered **Ir-2** in a solution of diethyl ether (2 mL); evaporation procedure; recovered **Ir-2** ready for the next reaction run

9. Recyclability of NHC-Ir(III) complex (**Ir-2**, Figure 9).
  a. After completion of the reaction, following the NHC-Ir(III) activation and mono-*N*-alkylation of unprotected amino acids (see previous sections), cool down the crude mixture to room temperature (20°C–23°C).
  b. Add diethyl ether (5 mL), and filter-off the mixture.
  c. Wash the solid (product **10**) with diethyl ether (5 mL × 2). Troubleshooting 4.
  d. Evaporate the combined ethereal solutions under reduced pressure in the rotavapor (40°C, 850 mbar).
  e. Use 2 mL of diethyl ether to transfer **Ir-2** to the reaction tube.
  f. Evaporate the solvent under vacuum and use the active catalyst **Ir-2** for the mono-*N-*alkylation of unprotected amino acids (next run).

## Expected outcomes

This protocol employs an efficient NHC-Ir(III) catalyst to achieve mono-*N*-alkylation of unprotected α-amino acids in a single step by using alcohols as alkylating agents. The applicability has been proven by synthesizing a large variety of *N*-alkylated amino acids with high retention of stereochemistry.^1^ The targeted modified amino acids were obtained in quantitative yields after a simple filtration without the need of derivatization or further purification techniques.

## Limitations

This protocol excludes amino acids containing a thiol group as cysteine (Cys), as well as basic amino acids such as lysine (Lys) and arginine (Arg). Another limitation of the methodology was encountered using secondary alcohols as alkylating agents.

## Troubleshooting

### Problem 1

The synthesis of the silver carbene **7** is a critical step in which the non-exposure to light and inert atmosphere are important (step 5).

### Potential solution

**Figure 10:**
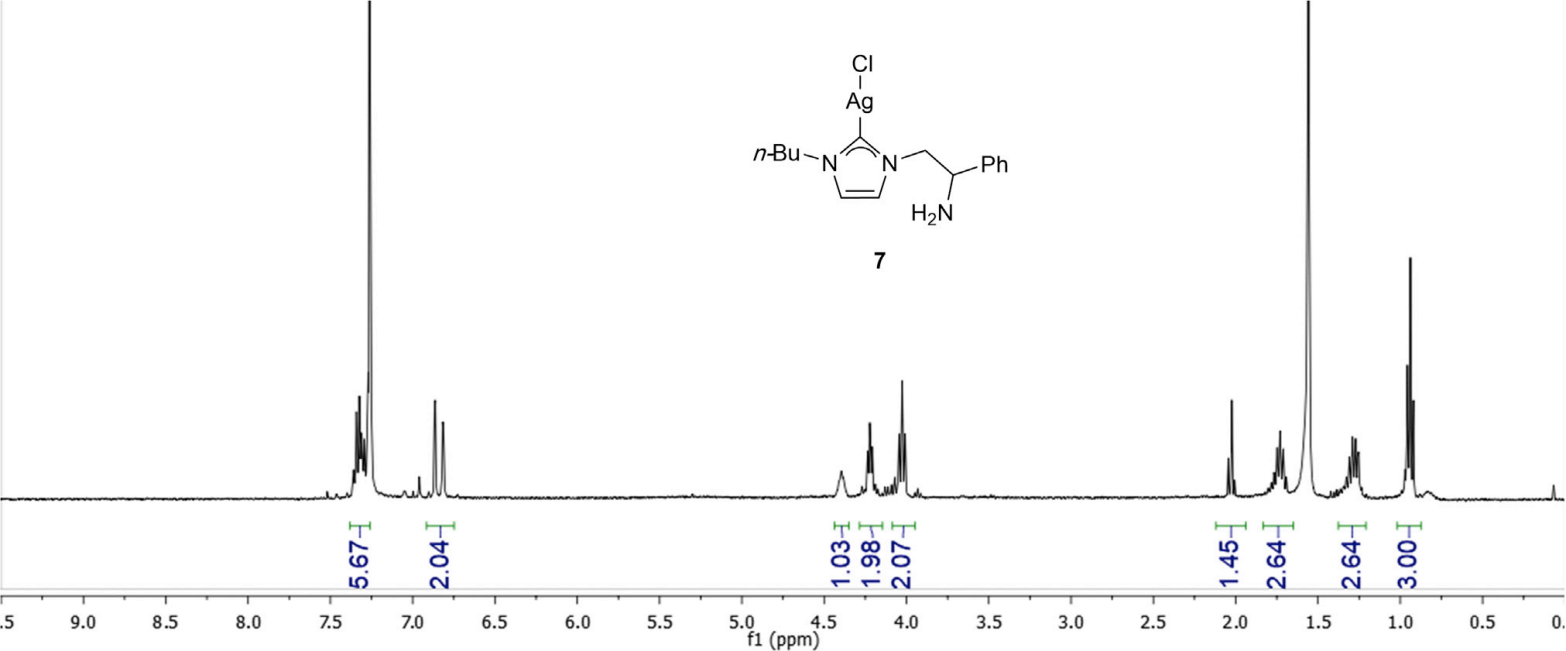
^1^H NMR (CDCl_3_) spectra of silver carbene **7**

• To ensure formation of silver carbene **7**, check crude by ^1^H NMR (CDCl_3_) before setting the next transmetalation step (Figure 10). Important to check the disappearance of the signal at 8.97 (s, 1H) from compound **6.**

### Problem 2

After collecting and evaporating the fractions corresponding to **Ir-1** catalyst, the complex is dissolved with the minimum amount of CH_2_Cl_2_ and precipitated with pentane. However sometimes after this, **Ir-1** should be precipitated/purified again repeating this precipitation process with pentane (step 6).

### Potential solution

**Figure 11:**
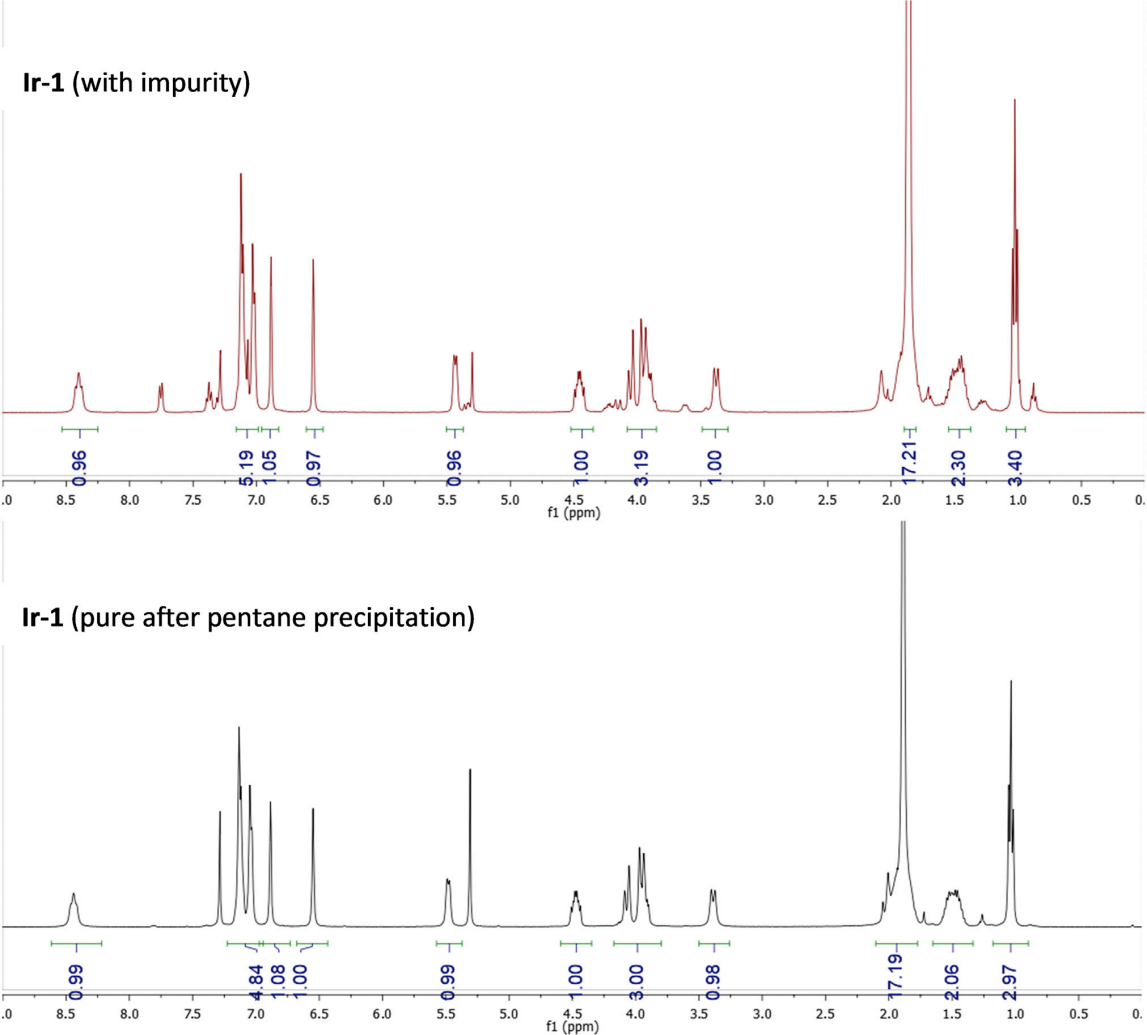
Comparison of purity of **Ir-1** before and after pentane precipitation step

• Here you can see and compare the ^1^H NMR (CDCl_3_) spectra of the impure **Ir-1** complex and the purified **Ir-1** (Figure 11).

### Problem 3

**Figure 12:**
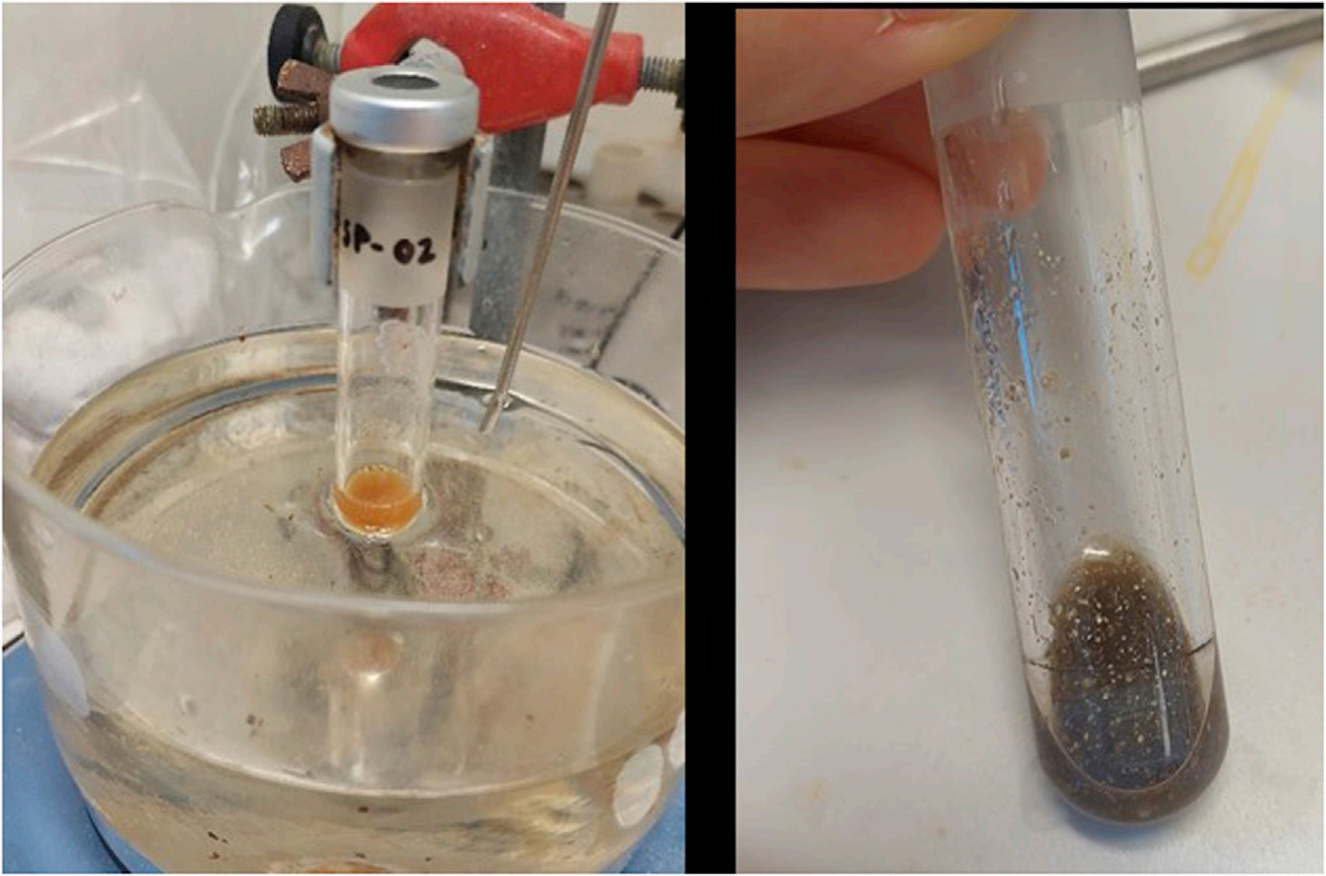
*N-*alkylation reaction appearance when the activation step fails

If the AgNTf_2_ salt is not properly kept or manipulated or if the activation step fails, the *N*-alkylation does not work (Figure 12) (step 8).

### Potential solution

• Keep AgNTf_2_ in a desiccator and avoid exposure to light. Do not keep it in the fridge/freezer.

### Problem 4

The reaction yield is lower than expected (i.e., quantitative) when using recycled **Ir-2** (step 9).

### Potential solution

• Product **10** solid was not washed efficiently. Further washing of **10** with diethyl ether is performed to ensure complete recovery of **Ir-2.**

## Resource availability

### Lead contact

Further information and requests for resources and reagents should be directed to and will be fulfilled by the lead contact Belén Martín-Matute (belen.martin.matute@su.se).

### Materials availability

This protocol did not generate new materials/products, all compounds have been described in the original article (Bermejo-López et al., 2022).^1^

### Data and code availability

The previous published article includes all datasets/code generated or analyzed during this protocol, see Bermejo-López et al.^1^
